# Post-infectious Painful Sensory Neuronopathy Following Giardia Infection Responsive to Intravenous Immunoglobulin Treatment

**DOI:** 10.7759/cureus.35053

**Published:** 2023-02-16

**Authors:** Ahmed Elrefaey, Anza B Memon

**Affiliations:** 1 Neurology, Ain Shams University, Faculty of Medicine, Cairo, EGY; 2 Neurology, John D. Dingell Veterans Affairs Medical Center, Detroit, USA

**Keywords:** giardia lamblia, intravenous immunoglobulins (ivig), ivig, neuronopathy, inflammatory neuropathy

## Abstract

Sensory neuronopathy is a rare pure sensory disorder with characteristic clinical features of early-onset ataxia and a multifocal distribution of non-length-dependent sensory deficits. Diabetes is the most common cause of length-dependent peripheral neuropathy. However, in acute to subacute presentations, conditions such as autoimmune diseases, paraneoplastic syndrome, exposure to toxins, and viral infection could be common etiologies. This report presents a patient with sensory neuronopathy following a *Giardia* infection. Gait disturbance, neuropathic pain, ataxia, and pseudoathetosis improved by varying degrees following the monthly maintenance of intravenous immunoglobulin (IVIG). An immune-mediated or direct pathogenic attack can explain the underlying pathogenesis behind this patient’s peripheral nerve dysfunction.

## Introduction

Sensory neuronopathy, also referred to as dorsal root ganglionopathy or sensory neuron disease, is a rare pure sensory disorder caused by injury to the dorsal root ganglion due to its fenestrated blood supply that permeates blood-borne molecules, antigens, or infecting agents [1]. The hallmarks of this peripheral nervous system dysfunction are early-onset ataxia coupled with multifocal sensory loss proximally and distally [2]. A broad list of etiologies can be grouped into metabolic and paraneoplastic syndromes, autoimmune diseases, drug-related toxins, and infections. Diabetes is the most common cause of length-dependent peripheral neuropathy. However, when confirmed to be of an infectious cause, sensory neuronopathy was traditionally exclusively observed in viral infections. Here, we report an unusual case of sensory neuronopathy following *Giardia* infection. A complete workup to rule out the common causes of this patient’s peripheral nerve dysfunction was unremarkable, leaving *Giardia* the most likely culprit.

## Case presentation

A 60-year-old female patient was admitted to our service, unable to walk and with unstable gait, ascending sensory deficits, severe neuropathic pain, and pseudoathetosis (proprioceptive deafferentation) four weeks into the diagnosis of *Giardia* infection. The patient and her daughter confirmed that she had stomach pain, vomiting, and diarrhea. She had a stool test positive for *Giardia* and received antibiotics at least twice. During her treatment, she started noticing difficulty with her balance and sensory symptoms, rapidly progressing and ascending up to her chest.

On examination, normal motor strength in all muscle groups (5/5 value in Medical Research Council {MRC} scale) was noted. However, the patient exhibited significantly reduced light-touch and pinprick sensations from the torso down in a non-length-dependent pattern, as well as marked loss of vibration and proprioception in all four extremities. She had a normal cranial nerve examination. Deep tendon reflexes were absent in the upper and lower extremities bilaterally.

An extensive neurological workup was unremarkable, including magnetic resonance imaging (MRI) of the brain and cervical, thoracic, and lumbar spine with and without contrast. A computed tomography (CT) of the chest, abdomen, and pelvis revealed no malignancy. A complete metabolic, autoimmune, infectious, neoplastic, and paraneoplastic workup yielded no positive results (Table 1).

**Table 1:** Laboratory and CSF results ACE, angiotensin-converting enzyme; Ab, antibody; AGNA-1, anti-glial nuclear antibody type 1; ANA, antinuclear antibody; ANNA, antineuronal nuclear antibody; C-ANCA, cytoplasmic antineutrophil cytoplasmic antibodies; CSF, cerebrospinal fluid; CRMP-5, collapsin response-mediator protein-5; dsDNA, double-stranded DNA; EBV, Epstein-Barr virus; GAD, glutamic acid decarboxylase; Hba1c, glycosylated hemoglobin; P-ANCA, perinuclear antineutrophil cytoplasmic antibodies; PCA, Purkinje cytoplasmic antibody; PCA-Tr: Purkinje cytoplasmic antibody titer; SS, Sjögren’s syndrome; ELISA, enzyme-linked immunosorbent assay; HSV, herpes simplex virus; Ig, immunoglobulin; PCR, polymerase chain reaction; TSH, thyroid-stimulating hormone; VDRL, venereal disease research laboratory; GM1, ganglioside monosialic acid 1; MAG, myelin-associated glycoprotein; TS-HDS, trisulfated heparin disaccharide idoA2SGlcNS-6S; Rpt, repeat; FGFR3; fibroblast growth factor receptor 3

	Values	Reference range/values
Laboratories		
Acetylcholine receptor antibody	<0.30	<0.30
ANA	Negative	Negative
C-ANCA	<1:20 titer	<1:20 titer
P-ANCA	<1:20 titer	<1:20 titer
dsDNA	Negative	Negative
GAD antibodies	<5 IU/mL	<5 IU/mL
Tissue transglutaminase, IgA	<2 IU/mL	<7 IU/mL
Tissue transglutaminase, IgG	<6 IU/mL	<7 IU/mL
SS A/Ro Ab	<1.0 ELISA units	<1.0 ELISA units
SS B/La Ab	<1.0 ELISA units	<1.0 ELISA units
Metabolic		
Sodium	135 mmol/L	135-145 mmol/L
Potassium	4 mmol/L	3.5-5.0 mmol/L
Chloride	104 mmol/L	98-111 mmol/L
CO_2_	25 mmol/L	21-35 mmol/L
Anion gap	6	3-13
Blood urea nitrogen	23 mg/dL	10-25 mg/dL
Creatinine	0.99 mg/dL	<1.03 mg/dL
Calcium	8.9 mg/dL	8.2-10.2 mg/dL
Hba1c	5.2%	<5.7%
Fasting glucose	103 mg/dL	<117 mg/dL
Vitamin B12	332 pg/ mL	180-810 pg/mL
Vitamin B6	14 μg/L	5-50 μg/L
Vitamin B1	64 μg/L	38-122 μg/L
TSH	2.03 μIU/mL	0.45-5.33 μIU/mL
Sensory (+/- motor) neuropathy panel		
IgG versus GM1	0	<2,000
IgG versus sulfatide	0	<3,000
IgM versus sulfatide	0	<3,000
IgM versus histone H3	0	<5,000
IgM versus GD1b	0	<3,000
IgM versus MAG	0	<3,000
IgM versus TS-HDS	0	<10,000
IgG versus FGFR3	0	<3,000
IgM versus GD1a	0	<2,000
Paraneoplastic autoantibody evaluation		
Amphiphysin Ab	Negative	<1.2 titer
AGNA-1	Negative	<1.2 titer
ANNA-1	Negative	<1.2 titer
ANNA-2	Negative	<1.2 titer
ANNA-3	Negative	<1.2 titer
CRMP-5-IgG	Negative	<1.2 titer
PCA-Tr	Negative	<1.2 titer
PCA-1	Negative	<1.2 titer
PCA-2	Negative	<1.2 titer
CSF results		
Tube number	4	
Volume	7 mL	
Color	Colorless	
Clarity	Clear	
Spun appearance	Clear	
Albumin, CSF		10-31 mg/dL
Glucose, CSF	62 mg/dL	40-80 mg/dL
Lactic acid, CSF	1.7 mmol/L	1.2-2.4 mmol/L
Protein, CSF	50.1 mg/dL	15-55 mg/dL
ACE, CSF	<5 U/L	≤15 U/L
RBC	<3 cu mm (H)	0/cu mm
Total nucleated cell count	5 cu mm	0-5 cu mm
Neutrophils, CSF	1%	0%-6%
Basophils	0%	0%
Eosinophils	0%	0%
Lymphocytes	63%	40%-80%
Macrophages	0%	0%
Monocytes	36%	15%-45%
Mononucleates	0 cu mm (L)	15%-45%
IgG, CSF		0.5-6.1 mg/dL
*Borrelia burgdorferi* Abs (EIA), CSF	0.07 LIV	≤0.99 LIV
VDRL, CSF	Rpt	
VDRL, CSF	Nonreactive	
West Nile IgG Abs, CSF	<1.30	
West Nile IgM Abs, CSF	<0.90	
West Nile virus, CSF	Negative	
Varicella zoster, PCR	Not detected	Not detected
Cytomegalovirus PCR, qualitative	Not detected	Not detected
EBV DNA, PCR	Not detected	Not detected
HSV-1 DNA	Not detected	Not detected
HSV-2 DNA	Not detected	Not detected
VDRL	Nonreactive	Nonreactive
IgG, serum	1,450 mg/dL	700-1,600 mg/dL
Albumin	3,005 mg/dL (L)	3,848-5,304 mg/dL
IgG index/CSF	0.6	0.3-0.7 ratio
Oligoclonal bands	Negative	Negative

Cerebrospinal fluid (CSF) appeared clear, with a normal protein level, leukocyte count, and IgG index, in addition to negative oligoclonal bands (Table 1). Electromyography and nerve conduction studies (EMG/NCS) showed a non-length-dependent sensory neuropathy/neuronopathy (Table 2).

**Table 2:** NCS and EMG at the time of initial presentation SNC, sensory nerve conductions; MNC, motor nerve conductions; NCS, nerve conduction studies; EMG, electromyography; EDB, extensor digitorum brevis; APB, abductor pollicis brevis; ADM, abductor digiti minimi; AH, abductor hallucis; MUAP, motor unit action potential; IA, insertional activity; PSW, positive sharp waves; Fib, fibrillation; Fasc, fasciculation; Dur, duration; Polys, polyphasia; N, normal

Nerve/sites	Recruitment site	Amplitude			Latency			Distance		Temperature	
		µV			ms			mm		°C	
		Right (R)	Left (L)	Reference	Right	Left	Reference	Right	Left	Right	Left
Median, digit II											
Wrist	Digit II	1.2		≥10.0	3.6		≤4.0	140		34	
Ulnar, digit V											
Wrist	Digit V	5.4		≥6.0	3.8		≤4.0	140		31.9	
Radial, wrist											
Forearm	Wrist	1.7		≥7.0	2.8		≤2.8	100		32.2	
Sural, ankle											
Calf	Ankle	3.5	4.5	≥4.0	3.7	3.2	≤4.5	140	140	31.9	32.9

Nerve/sites	Muscle	Amplitude			Latency			Distance		Velocity			Temperature	
		mV			ms			mm		m/s			°C	
		Right	Left	Reference	Right	Left	Reference	Right	Left	Right	Left	Reference	Right	Left
Median, APB														
Wrist	APB	9.6		≥4.2	3.9		≤4.4	80					32.9	
Elbow	APB	8.8			7.7			180		47		≥51	32.9	
Ulnar, ADM														
Wrist	ADM	12.1		≥7.9	3.2		≤3.7	80					32.1	
B. elbow	ADM	10.8			5.6			160		67		≥52	32.1	
A. elbow	ADM	9.8			8.5			110		38		≥43	32	
Peroneal, EDB														
Ankle	EDB	7.9	6.9	≥1.1	4.1	4.1	≤6.5	80	80				30.4	32.9
Popliteal fossa	EDB	5.5			10.4			270		43		≥38	30.6	
Tibial, AH														
Ankle	AH	13.7	14.1	≥5.3	5.5	5.3	≤6.1	80	80				31.2	32.7
Popliteal fossa	AH	10.7			13.0			360		48		≥39	31.1	

Nerve	Latency		
	ms		
	Right	Left	Reference
Median, APB	29.6		≤31.6
Ulnar, ADM	29.4		≤31.5
Tibial, AH	53.4	52.0	≤61.4
Peroneal, EDB	51.0	48.3	≤61.2

Nerve	Latency		
	ms		
	Right	Left	Reference
Median, APB	29.6		≤31.6
Ulnar, ADM	29.4		≤31.5
Tibial, AH	53.4	52.0	≤61.4
Peroneal, EDB	51.0	48.3	≤61.2

EMG summary table										
	Spontaneous					MUAP				
Muscle	IA	PSW	Fib	Fasc	Others	Effort	Recruitment	Amplitude	Dur	Polys
R. first dorsal interosseous	N	N	N	N	N	N	N	N	N	N
R. flexor carpi radialis	N	N	N	N	N	N	N	N	N	N
R. extensor digitorum communis	N	N	N	N	N	N	N	N	N	N
R. triceps brachii	N	N	N	N	N	N	N	N	N	N
R. biceps brachii	N	N	N	N	N	N	N	N	N	N
R. deltoid	N	N	N	N	N	N	N	N	N	N
R. tibialis anterior	N	N	N	N	N	N	N	N	N	N
R. medial gastrocnemius	N	N	N	N	N	N	N	N	N	N
R. flexor hallucis brevis	N	N	N	N	N	N	N	N	N	N
R. vastus lateralis	N	N	N	N	N	N	N	N	N	N
R. gluteus medius	N	N	N	N	N	N	N	N	N	N
R. lumbar paraspinals (low)	N	N	N	N	N					
R. thoracic paraspinals	N	N	N	N	N					

A right sural nerve biopsy revealed acute to subacute axonal neuropathy affecting 40%-50% of axons in all fascicles without regeneration and absent vasculitis or inflammation. A medial gastrocnemius muscle biopsy showed type 2 myofiber atrophy (Appendix).

The patient failed to improve on a five-day course of intravenous (IV) methylprednisolone (1 g) and improved slightly with plasmapheresis. The patient was discharged to an inpatient rehabilitation center and readmitted two weeks after discharge for worsening symptoms. Repeat EMG demonstrated worsening sensory responses in the upper extremities and interval development of mild denervation potentials in some selected muscles. Blink reflexes were also performed, which were normal on both sides (Table 3).

**Table 3:** Repeat NCS (including blink reflexes) and EMG two weeks later SNC, sensory nerve conductions; MNC, motor nerve conductions; NCS, nerve conduction study; EMG, electromyography; NR, not rated; EDB, extensor digitorum brevis; APB, abductor pollicis brevis; ADM, abductor digiti minimi; AH, abductor hallucis; MUAP, motor unit action potential; IA, insertional activity; PSW, positive sharp waves; Fib, fibrillation; Fasc, fasciculation; Dur, duration; Polys, polyphasia; N, normal

Nerve/sites	Recruitment site	Amplitude			Latency			Distance		Temperature	
		µV			ms			mm		°C	
		Right (R)	Left (L)	Reference	Right	Left	Reference	Right	Left	Right	Left
Median, digit II											
Wrist	Digit II	NR	NR	≥10.0	NR	NR	≤4.0	140	140	32.7	33.4
Ulnar, digit V											
Wrist	Digit V	NR	NR	≥6.0	NR	NR	≤4.0	140	140	32.2	32.9
Radial, wrist											
Forearm	Wrist	2.8		≥7.0	2.9		≤2.8	100		33.6	
Sural, ankle											
Calf	Ankle	NR		≥4.0	NR		≤4.5	140		32.7	

Nerve/sites	Muscle	Amplitude			Latency			Distance		Velocity			Temperature	
		mV			ms			mm		m/s			°C	
		Right	Left	Reference	Right	Left	Reference	Right	Left	Right	Left	Reference	Right	Left
Median, APB														
Wrist	APB	11.7		≥4.2	3.9		≤4.4	80					33	
Elbow	APB	10.4			7.6			180		49		≥51	33	
Ulnar, ADM														
Wrist	ADM	13.6		≥7.9	3.0		≤3.7	80					32.7	
B. elbow	ADM	13.2			6.1			170		54		≥52	32.6	
A. elbow	ADM	12.9			8.2			100		48		≥43	32.5	
Peroneal, EDB														
Ankle	EDB	4.5		≥1.1	3.5		≤6.5	80					32.5	
Popliteal fossa	EDB	4.0			10.2			290		43		≥38	32.5	
Tibial, AH														
Ankle	AH	14.0		≥5.3	4.8		≤6.1	80					32.5	
Popliteal fossa	AH	12.5			12.6			340		44		≥39	32.4	

Nerve	Latency		
	ms		
	Right	Left	Reference
Median, APB	28.1		≤31.6
Ulnar, ADM	28.8		≤31.5
Peroneal, EDB	48.0		≤61.2
Tibial, AH	49.5		≤61.4

Protocol/stimulation side	Ipsi R1	Ipsi R2	Contra R2
	ms	ms	ms
Supraorbital, orbicularis oculi (bilateral)			
Right	11.9	40.1	40.1
Left	11.4	39.5	42.8
Reference	13.0	41.0	44.0
R-L	0.5	0.6	2.8
Reference R-L	1.2	5.0	7.0

EMG summary table										
	Spontaneous					MUAP				
Muscle	IA	PSW	Fib	Fasc	Others	Effort	Recruitment	Amplitude	Dur	Polys
R. deltoid	N	N	N	N	N	N	N	N	N	N
R. triceps brachii	N	N	N	N	N	N	N	N	N	N
R. extensor indicis proprius	N	N	N	N	N	N	N	N	N	N
R. first dorsal interosseous	N	N	N	N	N	N	N	N	N	N
R. abductor pollicis brevis	N	N	N	N	N	N	N	N	N	N
R. vastus lateralis	N	N	N	N	N	N	N	N	N	N
R. tibialis anterior	N	N	N	N	N	N	N	N	N	N
R. medial gastrocnemius	N	N	N	N	N	N	N	N	N	N
R. peroneus longus	N	N	N	N	N	N	N	N	N	N
R. flexor hallucis brevis	N	N	N	N	N	N	N	N	N	N

Due to a significant loss of proprioception in the extremities, the patient lost the ability to fully activate her muscles. Intravenous immunoglobulin (IVIG) (2 g/kg) was administered over five days, and despite making partial improvement initially, the patient later experienced intermittent deterioration. Rituximab (1 g) was then received in two doses two weeks apart, and the patient regained the ability to walk with assistance, but a month later, it was also no longer effective. Response to steroid treatment was likewise poor. It was then decided to continue monthly maintenance of IVIG (1 g/kg). Three months later, the patient regained the ability to walk. Sensory ataxia and pseudoathetosis improved significantly, and neuropathic pain improved partially. The patient continues to be on multiple neuropathic pain medications (duloxetine 90 mg daily, pregabalin 200 mg three times/day, and amitriptyline 100 mg daily). Symptoms failed to improve on IV ketamine and lidocaine infusions.

## Discussion

Post-infectious neuropathy is thought to be caused by a direct pathogen invasion into the neuron or an indirect consequence of neurons cross-reacting with antibodies generated during an immune response to infection [3]. For example, Guillain-Barré syndrome (GBS), a common cause of acute post-infectious neuromuscular flaccid paralysis, is an autoimmune disease hypothesized to be induced via molecular mimicry, mainly as a sequela to *Campylobacter jejuni* enteritis, but has also been seen following other pathogenic infections such as Epstein-Barr virus, cytomegalovirus, and *Zika virus* [4].

Among plenty of subtypes of peripheral neuropathy is sensory neuronopathy, which is considered a rare entity with limited data on rates of incidence and prevalence available. Nevertheless, it has a distinctive clinical pattern of proprioceptive ataxia combined with non-length-dependent, asymmetrical sensory loss and, occasionally, more severe manifestations of pseudoathetosis and pseudoparesis. In clinical settings, comprehensive investigations are carried out to determine its underlying cause, and even then, around 50% of cases, such as the one we are presenting today, are rendered idiopathic [2].

In our case, we first suspected an underlying systemic neoplasm, refuted with a negative paraneoplastic panel (CSF and serum) and a lip biopsy. Other autoimmune diseases were ruled out with a negative complete immunologic paraneoplastic workup. Moreover, ganglionopathy induced by pyridoxine or chemotherapeutic agents was implausible, given that our patient was not affected by such supplements or drugs [4,1]. Viral infections were similarly eliminated with negative HIV and human T-cell leukemia virus type 1 (HTLV-1) results. Our thorough investigations have thus invalidated all possible causes of this patient’s unusual neurological findings, which led us to consider previously unconventional reasons such as *Giardia* [5].

## Conclusions

To date, giardiasis has not been considered among the etiologies of sensory neuronopathy, making this the first report of this rare finding. Giardiasis has not been classically associated with proprioceptive ataxia and asymmetrical sensory deficits, with its manifestations varying from an asymptomatic state to a severe malabsorption syndrome. This case thus adds value to future clinical practice by widening the scope of infectious etiologies of sensory neuronopathies to encompass parasitic infections such as *Giardia*.

The presented case report illustrates the occurrence of sensory neuropathy following a *Giardia* infection, extending the common etiologies of sensory neuronopathy to include such parasitic infection. Further research is warranted for this atypical association. Not only will this be instrumental in the rapid recognition of the signs and symptoms indicative of sensory neuronopathy in patients with giardiasis, but also it can pave the way for formulating appropriate management plans on time.

## Appendices

Operative diagnoses

Operation/Specimen

A. Nerve, right lower leg sural, biopsy B. Nerve, biopsy for electron microscopy C. Muscle for light microscopy, right leg gastrocnemius D. Muscle for histochemistry E. Muscle for electron microscopy

Pathological diagnosis

A, B, and C. Peripheral nerve, right sural, biopsy: moderate axonal neuropathy; see microscopic description and comment D, E, and F. Skeletal muscle, right gastrocnemius, biopsy: type 2 myofiber atrophy; see microscopic description and comment

Comment

Nerve Biopsy (A and B)

The biopsy shows an active, ongoing axonal neuropathy, with approximately 40%-50% axonal loss in all fascicles. The degree of active axonal degeneration and severity of the loss suggests an acute to subacute process. There is no evidence of significant axonal regeneration. There is no variation in the degree of axonal loss within and among the fascicles. No endoneurial or epineurial inflammation is seen, and there is no vasculitis.

The etiology of the neuropathy cannot be determined from the histologic examination, as is the case in the majority of axonal neuropathies. Both toxic/drug-related axonal neuropathies and acute motor-sensory axonal neuropathy may show similar findings histologically. Please correlate with clinical, laboratory, and electrophysiologic findings.

Muscle Biopsy (C, D, and E)

Type 2 myofiber atrophy is a reactive change frequently associated with disuse, prolonged steroid therapy, aging, and some systemic diseases, among other causes. No chronic neurogenic rearrangement (fiber-type grouping) is seen. The muscle is otherwise unremarkable. There is no evidence of a myopathic process, recent or remote, inflammatory or otherwise. No vasculitis is identified. Please correlate with clinical, laboratory, and electrophysiologic findings.

Microscopic description

Nerve Biopsy (A and B)

A. H&E and special stains: An H&E stain and special stains/immunostains were performed on block A1 (formalin-fixed tissue) with adequate controls. H&E sections contain 24 fascicles of peripheral nerve. Many myelin ovoids and digestion chambers were seen. The endoneurial and perineurial microvasculature is unremarkable. No inflammatory infiltrate is noted. There was no evidence of vasculitis.

Special stains: There are no amyloid deposits identified on Congo red stain. Trichrome stain and Luxol fast blue stains highlight the patchy loss of myelin staining. No iron deposition is seen.

Immunohistochemistry: Neurofilament stain highlights axonal architecture with the patchy loss of axonal density. No giant axons are seen. Cluster of differentiation 163 (CD163) immunostain highlights many immunoreactive cells in the endoneurium. CD3 immunostain highlights few scattered T-cells in the endoneurium and perineurium. B-cells (CD20-positive) are virtually absent.

B. Plastic-embedded material: Thin paragon-stained sections obtained from two Epon-embedded blocks were examined with a light microscope.

The sample contains seven fascicles of peripheral nerve. There is marked axonal loss (approximately 40%-50%). The axonal loss appears to be predominantly myelinated fibers. Many acutely degenerating axons are noted. The remaining axons have normal myelin sheath thickness. No regenerating clusters or onion bulbs are seen. The fascicles are uniformly affected. No giant axons or myelin abnormalities are observed. No abnormal deposits are identified. The endoneurial and perineurial microvasculature is normal. There is no inflammatory infiltrate and no vasculitis.

Muscle Biopsy (C, D, and E)

H&E, enzyme histochemistry, and special stains: H&E, Congo red, and all immunostains except major histocompatibility complex 1 (MHC-1) are performed on separate slides from block C1 (fixed paraffin-embedded tissue). All other histochemical stains and MHC-1 immunostain were performed on separate slides from block D1 (frozen tissue).

H&E-stained paraffin and cryostat sections show moderate myofiber size variability with scattered small-angulated myofibers. There is no endomysial, perimysial, or epimysial inflammation. No vasculitis is seen. No necrotic or regenerating myofibers are seen. Other myopathic features (interstitial fibrosis or increased internal nuclei) are absent. CD163 immunostain highlights macrophages in the interstitium, as well as perimysium. CD3 immunostain shows few scattered immunoreactive cells in the endomysium and perimysium. B-lymphocytes (CD20-positive) are virtually absent. MHC-1 immunostain shows no significant upregulation and highlights the normal endomysial capillary bed.

Modified trichrome-stained sections demonstrate a normal punctate sarcoplasmic staining pattern without the evidence of ragged red fibers, rimmed vacuoles, or other abnormal sarcoplasmic accumulations. ATPase preparations at pH 4.3, 4.6, and 9.5 show marked predominance of type 1 myofibers. No definite fiber-type grouping or group atrophy is noted. The small-angulated myofibers are mostly type 2. Nicotinamide adenine dinucleotide hydrogen (NADH)-stained preparation reveals a normal sarcoplasmic staining pattern with no disruption of the intermyofibrillar network. No target fibers are seen. Esterase-stained preparation highlights the occasional motor end plate. Periodic acid-Schiff (PAS)-stained preparation reveals no increase in glycogen stores. There is no evidence of an increase in neutral lipid store in oil red O preparations. Alkaline phosphatase activity is not increased. Myophosphorylase and adenylate deaminase stains confirm the presence of these enzymes in the muscle fibers. No cyclooxygenase (COX)-negative fibers with abnormal mitochondria (succinate dehydrogenase {SDH}/phenazine methosulfate {PMS} and COX/SDH) are noted. Congo red is negative for amyloid deposition.

Thin paragon-stained sections obtained from three Epon-embedded blocks were examined with a light microscope. There is no evidence of abnormal structures or deposits. The sections show similar findings to the paraffin-embedded and frozen tissue described above.
